# Inducible UCP1 silencing: A lentiviral RNA-interference approach to quantify the contribution of beige fat to energy homeostasis

**DOI:** 10.1371/journal.pone.0223987

**Published:** 2019-11-21

**Authors:** Nicole Wen Mun Khor, Michael M. Swarbrick, Jenny E. Gunton

**Affiliations:** 1 The Westmead Institute for Medical Research, Westmead, Sydney, Australia; 2 Garvan Institute of Medical Research, Darlinghurst, Sydney, Australia; 3 Faculty of Medicine, University of New South Wales, Sydney, Australia; 4 Faculty of Medicine and Health, The University of Sydney, Sydney, Australia; State University of Rio de Janeiro, BRAZIL

## Abstract

Energy consuming, heat-producing beige adipocytes, located in classic white adipose tissue (WAT), hold promise for the treatment of obesity. Few reports have quantitatively assessed the contribution of browned 'WAT' to energy expenditure. There is a need for methods to examine beige-fat thermogenesis, independently of classical brown fat. The aim of this study is to optimize an inducible lentiviral shRNA to conditionally knock-down *Ucp1* and assess the effects on 'browned' WAT. Primary adipocytes from mouse inguinal WAT converted into thermogenic adipocytes when stimulated with β-adrenergic agonist and thiazolidinedione. There was increased UCP1 protein and importantly increases in various indicators of mitochondrial bioenergetics. Next, we determined optimal transfection conditions for the UCP1-shRNA lentiviral system and subsequently applied this to 'browned' WAT. UCP1 knockdown decreased the brown/beige-fat gene profile and decreased mitochondrial respiration. In summary, this study optimizes lentiviral UCP1-shRNA technology in *vitro*. This technique could be applied to inguinal fat depots *in vivo*. This would allow investigation of contribution of depots to whole-body metabolism to help elucidate the physiological relevance of beige fat.

## Introduction

For the first time in human history the global burden of overnutrition outpaces that due to undernutrition. Worldwide overweight and obesity accounts for at least 2.8 million deaths each year and 35.8 million disability-adjusted life years [1]. Obesity is a pathological state characterized by accumulation of excess adipose tissue and lipids. It increases the risk of glucose intolerance, hypertension and dyslipidemia. Over time, obesity contributes to cardiovascular disease, type 2 diabetes mellitus and many cancers. Effective treatment regimens are scarce and new therapeutic targets are needed. Given its central role in energy and nutrient homeostasis, many have looked towards adipose tissue for a solution.

Traditionally adipose tissue was divided into two distinct types. Unilocular white adipose tissues (WAT) make up the bulk of fat and primarily function to store energy. Conversely brown adipose tissue (BAT) is highly specialised for thermogenesis as a defence against cold, a property conferred by uncoupling protein-1 (UCP1). UCP1 dissipates the mitochondrial membrane proton gradient, uncoupling fuel oxidation from ATP synthesis and converting nutrient-derived energy into heat [2, 3]. Chronic activation stimulates BAT proliferation and differentiation as well as lipolysis, releasing free fatty acids as fuel for UCP1-mediated thermogenesis [4].

BAT is known to cluster at the interscapular and perirenal regions of newborn infants [5]. However, in 2009 positron emission tomography (PET) with [18F]-fluorodeoxyglucose (FDG) studies identified BAT in the supraclavicular, mediastinal and thoracic regions of cancer patients. Subsequent biopsies confirmed humans adults possess BAT containing bona-fide UCP1+ cells [6]. Although BAT is small in volume, its potential thermogenic contribution is significant. BAT activity has been predicted to account for 2.7–5% of basal metabolic rate (BMR) in humans, which dissipates energy equivalent to 4kg a year [6, 7]. Some reports suggest maximal activation of BAT could increase daily resting energy expenditure (EE) by up to 20% [8].

Independently of BAT, cells possessing many of the anatomical and molecular features of brown adipocytes have been identified within WAT depots. These are called beige or brite adipocytes. Compared to classical BAT, beige cells express less basal UCP1 and show lower uncoupled respiration [4]. However upon stimulation with prolonged cold exposure or adrenergic signalling, they activate a thermogenic program and elevate UCP1 to levels more like BAT [9]. This beige fat has obvious therapeutic potential for obesity.

The specific contribution of browned WAT to energy expenditure and fuel metabolism, independently of BAT has not been determined. Studies ablating UCP1 cells or mutating UCP1 illustrate the overall importance of brown and beige fat yet the individual contributions of these two distinct set of cells have been impossible to elucidate. However, few reports have provided quantitative assessments of the effect of WAT browning on its thermogenic capacity. Recently Cohen and Levy [10] showed adipocyte-specific deletion of the co-regulatory protein PRDM16 causes minimal effects on classical BAT but markedly inhibited beige adipocyte function.

The aim of this study is to optimise a regulatable lentiviral shRNA platform for conditional UCP1 deletion. The research tool serves as a means to quantitate the metabolic contribution of beige fat independently of BAT. Lentiviruses are positive-strand RNA viruses that can transduce both dividing and non-dividing cells to stably integrate their genomes into host cell chromosomes [11]. Here the vector is transcribed to deliver UCP1-shRNA—stem loop RNA structures targeted to silence *Ucp1*-gene expression via RNA interference. This system permits regulation of shRNA expression with potent induction in the presence of doxycycline [12].

## Materials and methods

### Isolation and culture of primary adipose cells

Male mice (7–10 weeks old, C57BL/6J, from Australian Bioresources, Moss Vale, NSW) were used to obtain primary adipocytes. This study, including all animal studies and procedures, was approved by the Western Sydney Local Health District Animal Ethics Committees (WSLHD AEC, approval #4222.06.14 Obesity and adipose tissue). Mice were anaesthetised using isoflurane and euthanised under anaesthetic by cervical dislocation. Inguinal fat pads were dissected, minced and digested in a digestion buffer for 45 minutes at 37°C, shaking at 100rpm. The digestion buffer consisted of phosphate buffered saline (PBS) containing 2.4U/mL Dispase II and 1.5U/mL collagenase D (both from Roche Diagnostics, IN, USA). Digested tissue was then filtered through a 100-μm cell strainer (Thermo-Fisher Scientific, CA, USA) and the flow-through centrifuged at 600*g* for 5 minutes to pellet the SV cells. Cells were resuspended in complete SV culture medium consisting of: DMEM/F12 + glutamax (Thermo-Fisher, CA, USA), Penicillin-Streptomycin (Thermo-Fisher) and 10% foetal bovine serum (GE Healthcare Hyclone, Utah, US). This mixture was filtered using a 40-μm cell strainer to remove clumps and large adipocytes. Adipocyte cell number was calculated and plated onto cell culture plates. Cells were maintained in an incubator at 37°C under 5%CO_2_/95% O_2_. Cell medium was changed every second day until adipocytes reached confluence.

For adipocyte differentiation assays, confluent cultures were exposed to an adipogenic cocktail containing SV culture medium plus 5μg/mL insulin (Torrent Pharma, Gurajat, India), 1μM dexamethasone, 0.5mM isobutylmethylxanthine and 1μM rosiglitazone (compounds from Sigma-Aldrich, MO, USA). Forty-eight hours after induction cells were maintained in SV media containing 5μg/mL insulin. For treated groups, drugs were delivered to a final concentration of 1μM rosiglitazone and 100nM CL316,243 (Sigma-Aldrich, MO, USA). Media was changed every two days for rosiglitazone and daily for CL316, 243.

### Seahorse analyzer—O_2_ consumption analysis

Cell seeding was performed in Seahorse 24-well XF Cell culture from Seahorse Bioscience, Billerica, MA, USA. Adipocytes were harvested and re-suspended to concentration of 15,000 cells to be seeded in 100μL of growth medium. Plates were placed in incubator and monitored for adherence. 150μL of growth medium was added after 4–5 hours to each well bringing the total volume to 250μL.

An XF24 extracellular flux analyser was used to determine mitochondrial function (XF24, Seahorse Bioscience). In order to analyse respirometry the following substances were added [concentration injected: final concentration in well]. Stage I: oligomycin (6μM:1μM), stage II: FCCP–Carbonyl cyanide 4-(trifuormethoxy) phenylhydrazone (35μM:5μM), stage III: (200μM: 25μM); stage IV: Rotenone [9 μM:1 μM] (Sigma-Aldrich, Sydney, Australia). Two step FCCP dose was performed as differential sensitivity to FCCP in WAT and BAT has been observed.

### Short hairpin RNA experiments

#### Determining functional titre

The lentivirus protocol was optimised by transfecting adipocytes with control lentivirus expressing enhanced green fluorescence protein (eGFP) and shRNA directed against GAPDH. A range of multiplicity of infection (MOI) was tested including 1, 5, 10, 25 and 50. In 96-plate wells transduction medium DMEM (Thermo-fisher Scientific), polybrene (final concentration 8μg/ml; Sigma-Aldrich) and inducible lentivirus encoding shRNA-GAPDH [glyceraldehyde-3-phosphate dehydrogenase] (GE Dharmacon, Millennium Science, Melbourne, VIC, Australia) was added, bringing each well to a total volume of 25μL. Six hours later, 100μL of DMEM was added to each well. Cells were replenished with medium containing freshly dissolved 700ng/ml doxycycline (MP Biomedicals, Solon-Ohio, USA) 24 hours later to induce shRNA. Forty-eight hours later, eGFP expression was assessed using a high-quality fluorescence microscope (Zeiss Axiovert 200M, Carl Zeiss, Gottingen, Germany).

#### Lentiviral shRNA-UCP1 on CL316,243 treated cells

Adipocytes were plated onto 96-well plates and grown to 70–80% confluence. Cells were transfected with lentivirus encoding shRNA targeted against UCP1 (GE Dharmacon) at a MOI of 50 (Day 1). Each well consisted of 50μL of lentivirus plus 5.5μL of polybrene (final concentration 8μg/ml). At Day 3 base cell culture medium was changed to medium containing: (A) SV media plus insulin (B) SV media plus insulin plus doxycycline (700ng/mL), (C) SV media plus insulin plus CL (100nM) or (D) SV media plus insulin + CL + doxycycline. Conditions are summarized in Table 1. Forty-eight hours later GFP expression and cell-morphology was assessed, followed by RNA extraction gene expression analysis.

**Table 1:** Groups UCP1-shRNA in CL316, 243 treated adipocytes.

Row	Treatment	Doxycycline	Lentivirus
**A**	Control (SV + Insulin)	-	+
**B**	Control	+	+
**C**	CL (SV + Insulin + CL)	-	+
**D**	CL	+	+

#### Lentiviral UCP1-shRNA on Rosiglitazone treated cells

The above protocol was conducted for Rosiglitazone treated cells in Seahorse 24-well XF Cell culture microplates (Seahorse Bioscience, Table 2). Forty-eight hours after induction of virus by Doxycycline, cells underwent a mitochondrial stress test using the Seahorse XF Analyzer.

**Table 2:** Groups—UCP1-shRNA in Rosiglitazone treated adipocytes.

Row	Treatment	Doxycycline	Lentivirus
**1**	Control	-	-
**2**	Control	+	-
**3**	Rosiglitazone	-	-
**4**	Rosiglitazone	+	-
**5**	Rosiglitazone	-	+
**6**	Rosiglitazone	+	+

### RNA extraction and quantitative real-time PCR

Total RNA was extracted using a Tri reagent (Sigma-Aldrich, MO, USA) and RNA concentration/quality was assessed by a NanoDrop spectrophotometer. cDNA was generated using the Maxima First Strand cDNA Synthesis Kit for RT-qPCR (Thermo, Scientific, MA, USA) as per manufacturer's instructions. Gene expression (RNA 18S, CIDEA, leptin, PCG-1α, TBP, TBX 1, UCP 1) was measured using Power SYBR green master mix (Applied Biosystems, Scoresby, Australia). *18S* was used as an endogenous reference house-keeping gene. Following the reaction, fold change was calculated using the 2ΔΔCT method.

### Cell lysate and protein quantification

Tissue extracts were lysed in RIPA buffer [1% NP-40, 0.1% SDS, 150mM NaCl, 50mM Tris-HCl (pH 7.5), 0.5% Na-deoxycholate, 2mM EDTA and 50mM NaF], ingredients from Astral Scientific, NSW, Australia. Protease inhibitor cocktail buffer (Roche-Diagnostics, IN, USA) was freshly added to 50ml of RIPA buffer. Protein content of each extract was determined by DC protein assay kit (Bio-Rad Laboratories, California, USA) and absorbance measured at 595 nm using the Viktor Plate Reader X24 (Perkin-Elmer, Waltham, MA, USA).

### Immunoblotting

Equal amounts of protein sample were prepared in SDS-sample buffer and heated for 10 minutes at 100°C. Cleared lysates were electrophoresed in 10% polyacrylamide gels and transferred to PVDF membranes. Membranes were blocked in TBST with 5% skim milk powder to prevent non-specific binding. Membranes were probed with rabbit polyclonal anti-UCP1 1:1000 (#ab23841, Abcam, Cambridge, UK), and anti-rabbit IgG HRP-linked antibody 1:5000 (#7074, Cell Signalling Technology, Qld, Australia). Density of protein signal was normalized to that of the internal control, 14-3-3 1:2000 (Sc-629 Santa Cruz, CA, USA). Protein expression was visualized using enhanced chemiluminescence, and densitometry of UCP1 and 14-3-3 bands was performed using ImageJ software (NIH, Bethesda, MD, USA).

### Statistical analysis

Data are presented as means ± SEM unless indicated otherwise where *p<0.05, **p<0.01; ***p<0.001, ****p<0.0001. Statistical significance was determined by one way ANOVA using Graphpad Prism 6.0 (GraphPad Software, San Diego, CA). Where multiple comparisons were made, Tukey's multiple comparison test was used.

## Results

### Activation of UCP1-mediated thermogenesis

#### CL316, 243 and Rosiglitazone induce unilocular-to-multilocular transformation and increased UCP1 protein expression

**Fig 1:**
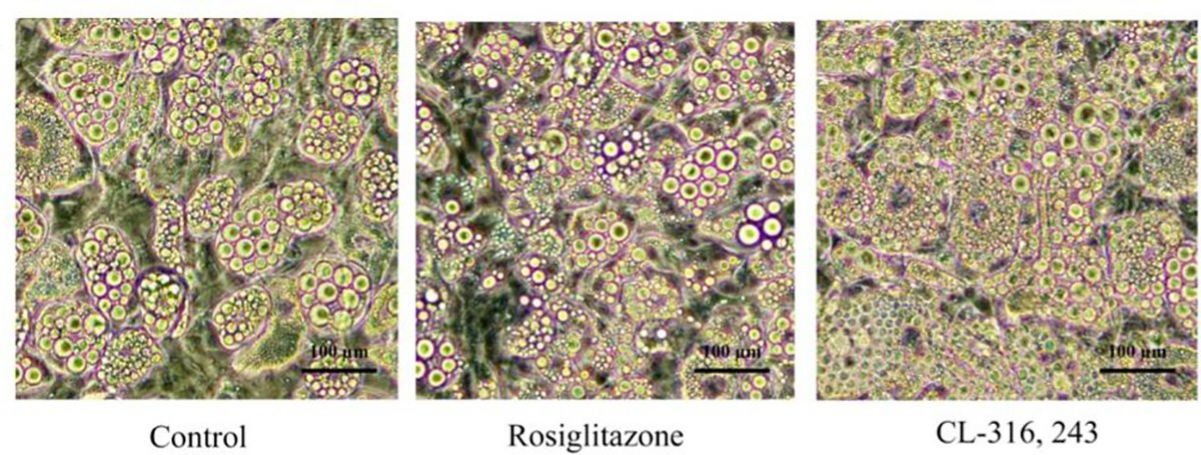
Morphology of adipocytes derived from control, Rosiglitazone and CL316, 243 treated cells, 10 days *in vitro* (see S2 Fig for differentiation of adipocytes post-treatment across various time points).

**Fig 2:**
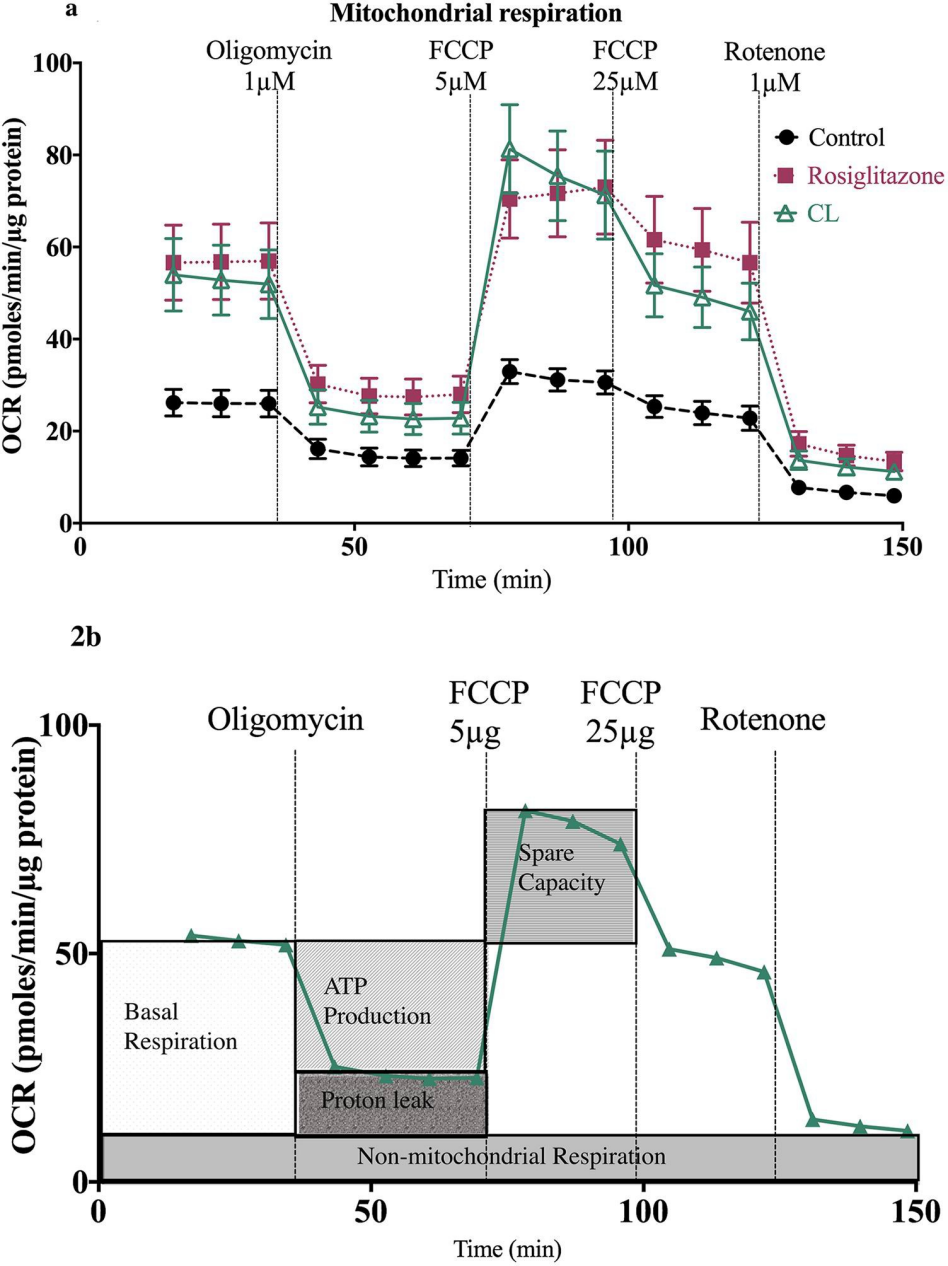
Beige adipocytes demonstrate increased oxygen consumption rates in the presence of Rosiglitazone and CL316, 253. (a) Time course of oxygen consumption rate (OCR) of differentiated beige adipocytes under control conditions and in the presence of Rosiglitazone and CL316, 243, 8–10 days *in vitro*. (b) The schematic illustrates the proportion of basal respiration, ATP production, proton leak respiration (after Oligomycin), maximal substrate oxidation (after FCCP 5μg) and non-mitochondrial respiration (Rotenone). Data represented as mean ± SEM; n = 51; analysed by one-way ANOVA. *p<0.05, **p<0.01; ***p<0.001, ****p<0.0001 vs. control.

Adipocytes from C57BL/6J mice were differentiated and cultured under adipogenic conditions with the addition of Rosiglitazone and CL316, 243. As expected, standard light microscopy reveals multilocular fat droplets, characteristic of classical BAT in treated cells (Fig 1). Beige adipocytes from C57BL/6J mice under mitochondrial stress test demonstrate increased oxygen consumption rates in the presence of Rosiglitazone and CL316, 253 (Fig 2A and 2B).

#### Browned adipocytes exhibit increased UCP1-mediated thermogenesis and increased mitochondrial bioenergetics

**Fig 3:**
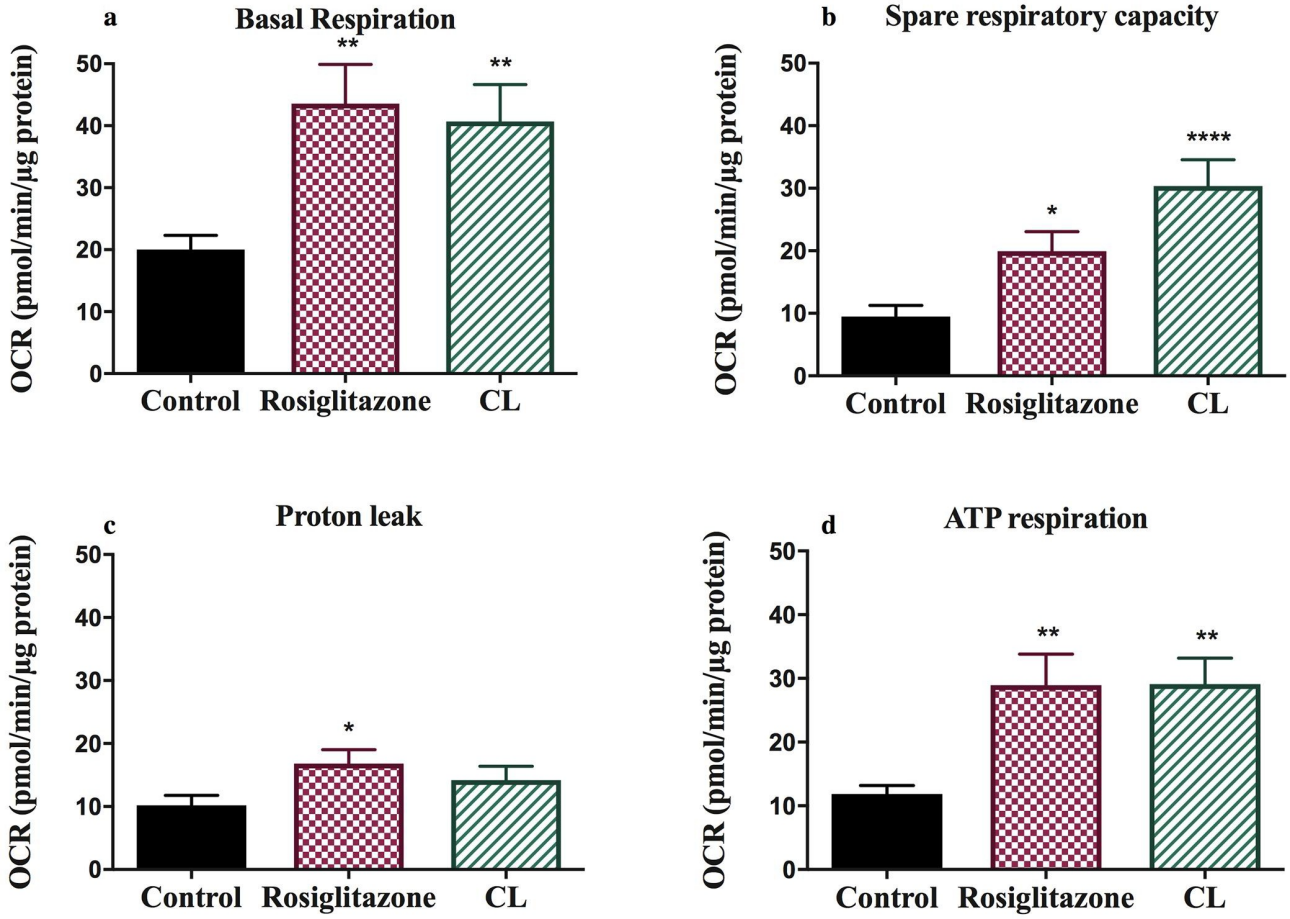
Derived parameters of respiration (proton leak, ATP respiration, basal respiration and space respiratory capacity) across control, Rosiglitazone and CL316, 243 treated adipocytes. Data represented as mean ± SEM; n = 51; analysed by one-way ANOVA. *p<0.05, **p<0.01; ***p<0.001, ****p<0.0001 vs. control.

A salient feature of chronic β3 activation is pronounced remodelling of WAT involving mitochondrial biogenesis and elevation of metabolic rate [13]. Furthermore thiazolidinediones such as Rosiglitazone induce *Ucp1* gene expression and increase mitochondrial biogenesis [14]. Here we attempt to quantify changes in mitochondrial function using a microplate based extracellular flux analyser. Respiration rate in the presence of oligomycin is a direct measure of the proton leak rate across the mitochondrial membrane *in situ*. Proton leak kinetics is mainly mediated by UCP1. Rosiglitazone increased UCP1-mediated respiration by 37% (p<0.05) and CL316, 243 by 65% (p = 0.30). Furthermore ATP respiration increased by approximately 2.5 fold in both rosiglitazone (p<0.001) and CL316, 243 (p<0.001) treated adipocytes (Fig 3).

Basal respiration is usually controlled by ATP turnover and partly by substrate oxidation and proton leak [15]. Consistent with the above results, there was a 2.17 (p<0.001) and 2.03 fold (p<0.001) increase in ATP respiration in rosiglitazone and CL316, 243 treated cells respectively. However there is capacity of treated adipocytes to increase energy consumption even further as reflected in the spare respiratory capacity—the amount of extra energy that can be produced in response to stress or increased workload [15]. Compared to control rosiglitazone increased spare respiratory capacity 2.1 fold (p<0.05) and CL316, 243 by 3.2 fold (p<0.0001), (Fig 3).

**Fig 4:**
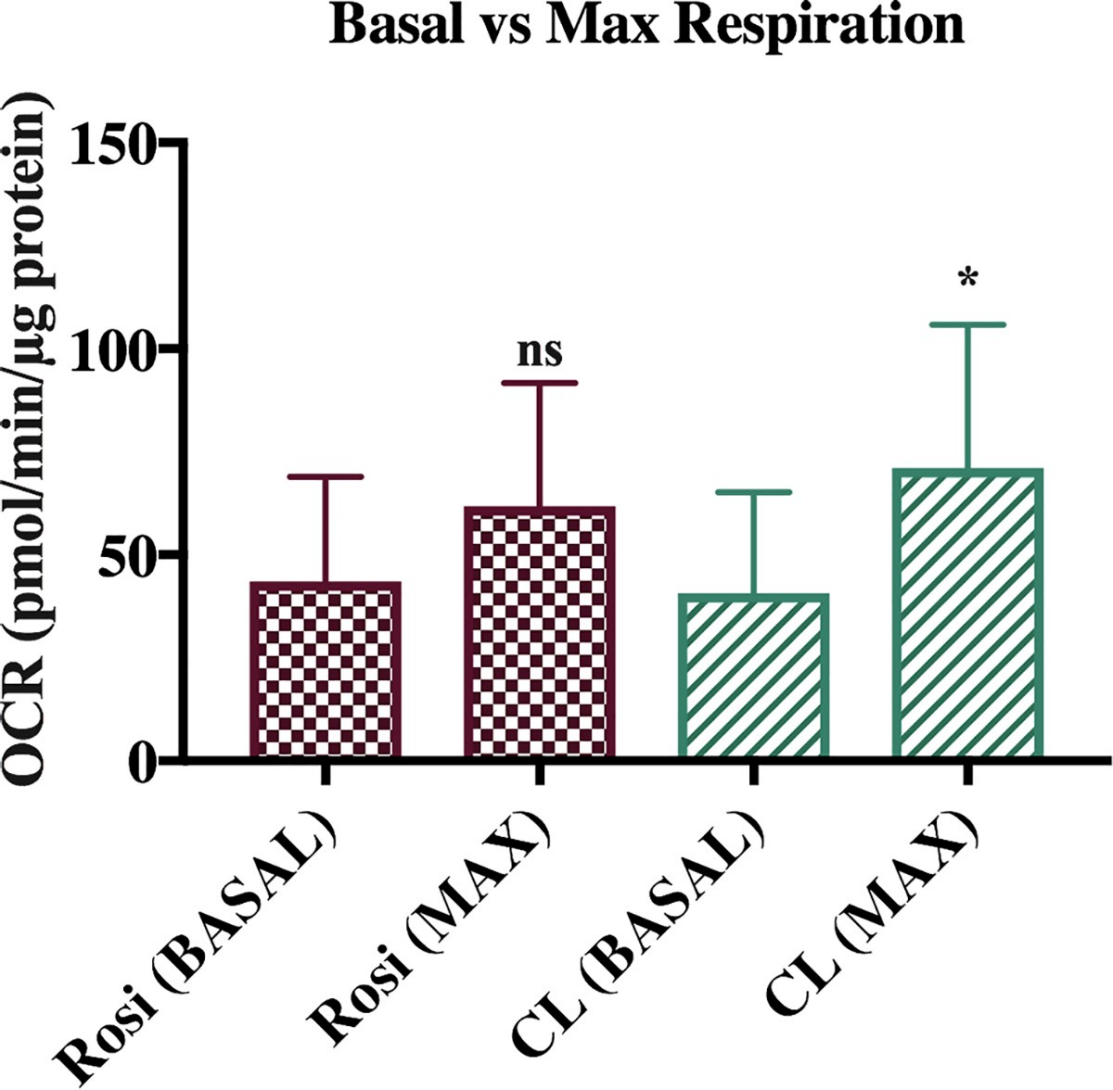
Basal vs maximal respiration in Rosiglitazone (n = 32) and CL 316, 243 (n = 34) treated cells. Data represented as mean ± SEM; analysed by one-way ANOVA. *p<0.05, **p<0.01; ***p<0.001, ****p<0.0001 vs. control.

The potential of treated cells to increase expenditure is clearly illustrated by examining maximal respiration. The addition of FCCP permeabilizes the inner mitochondrial membrane to reveal the maximal respiratory capacity. Whilst previous reports recommend 20μM, we have found injection of 5μM of FCCP elicited maximal respiratory capacity [16]. When comparing basal respiration to maximal respiration in Rosiglitazone and CL316, 243 treated cells (Fig 4), we see a 1.4 and 1.75 fold increase in OCR for rosiglitazone and CL316, 243 respectively.

#### Thermogenic agents enhance glycolytic capacity

**Fig 5:**
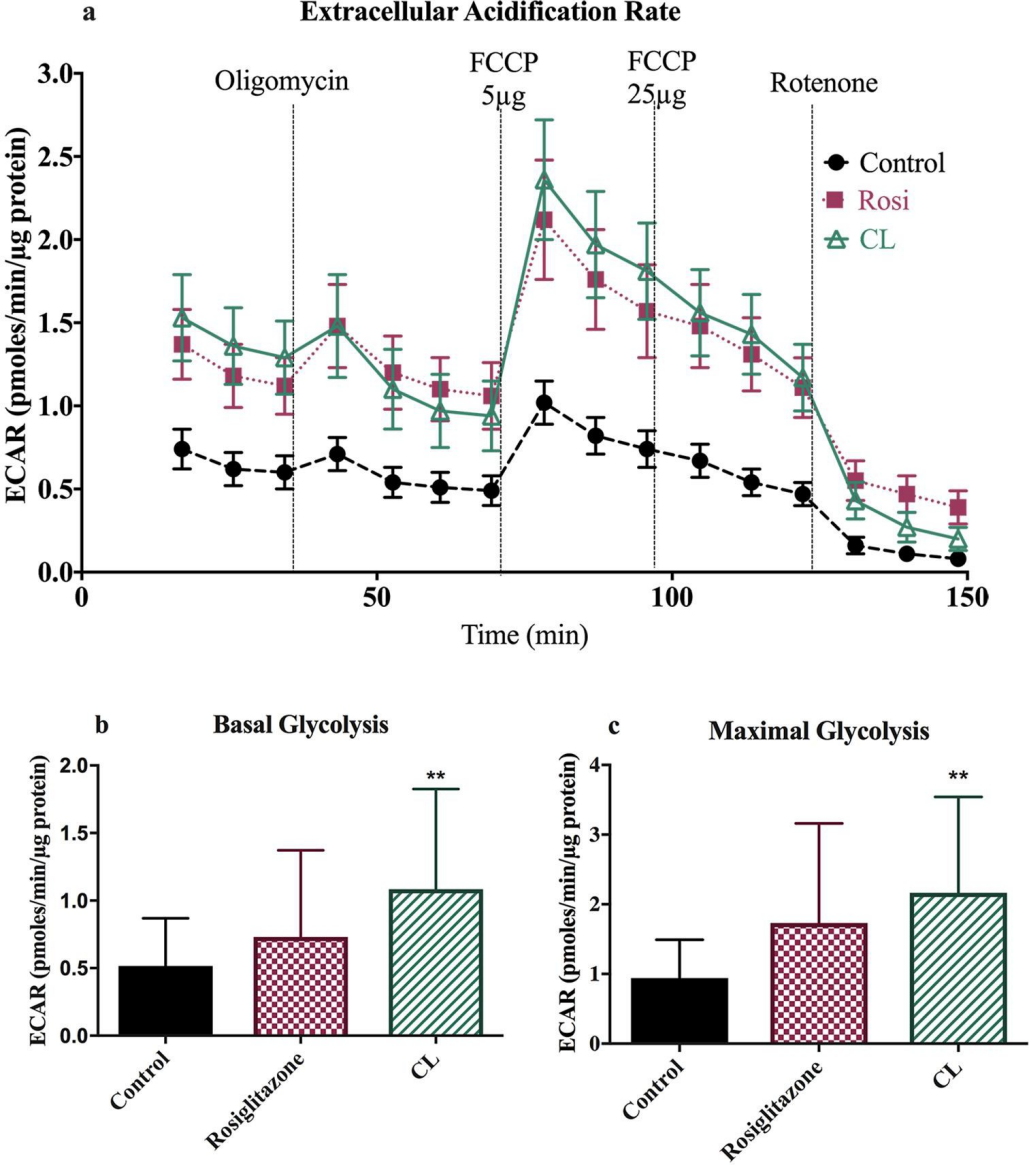
Extracellular Acidifaction Rate (ECAR) in control, rosiglitazone and CL316, 243 treated adipocytes. (a) Time course of ECAR of differentiated beige adipocytes (b and c) Basal and maximal glycolysis (after FCCP 5μg). Data are represented as mean ± SEM; n = 51.*p<0.05, **p<0.01; ***p<0.001, ****p<0.0001 vs. control, one-way ANOVA.

Extracellular acidification rate measures changes in extracellular pH as a response to lactate formation which is an indicator of glycolysis. Compared to control, basal glycolysis increased by 41% (p = 0.48) in rosiglitazone and 109% (p = 0.008) in CL316,243 treated cells. Maximal glycolysis increased even further; 84% for rosiglitazone (p = 0.08) and 130% (p = 0.003) for CL316,243 treated adipocytes (Fig 5).

The magnitude for the changes are small yet indicate increased capacity for glycolysis in treated cells. The release of lactate and pyruvate from BAT due to thermogenic stimulation have been shown to account for 33% of glucose uptake in rats [17] and thus these values may represent a larger proportion of glycolysis *in vivo*. Collectively these results indicate thermogenic drugs induce changes in a variety of metabolic pathways—an increased capacity for uncoupled respiration as well as glycolysis.

### Effect of lentiviral UCP-1 shRNA on adipocytes

#### Optimal infection conditions for mature adipocytes

The above results confirm that beige adipocyte thermogenesis can be simulated *in vitro*, demonstrated on a morphological, functional and protein level. The next step was to test whether the inducible lentiviral UCP1-shRNA system can effectively inhibit UCP1 expression and function. This firstly requires determination of optimal transfection conditions of mature adipocytes.

**Fig 6:**
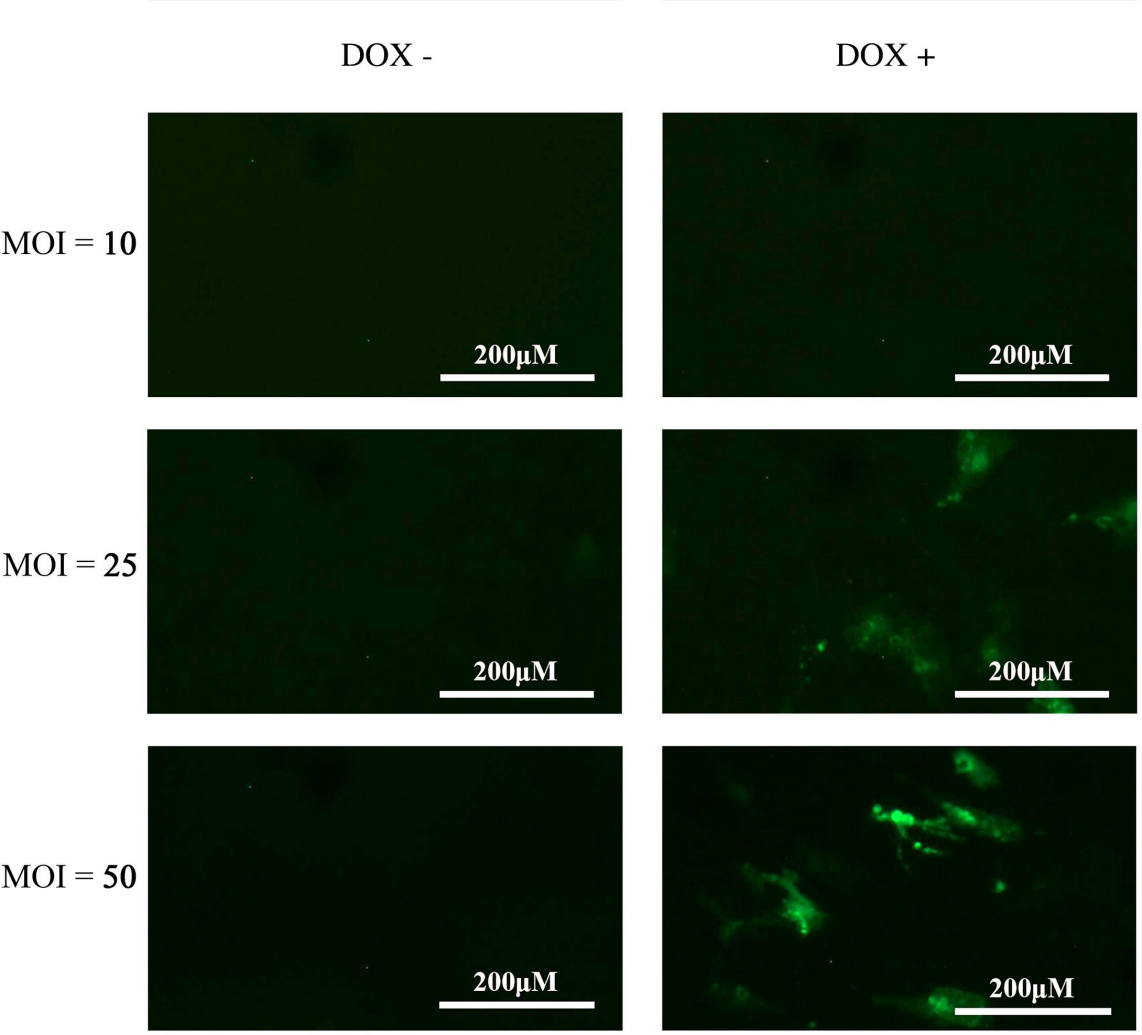
Determining optimal multiplicity of infection (MOI) for transduction of lentiviral shRNA genome, induced with Doxycycline (DOX). Control lentivirus expressed green fluorescent protein (GFP) and shRNA directed against GAPDH. Transfected cells are shown from 10 MOI and up, as there was no significant GFP below that level.

Expression of fluorescence in doxycycline treated adipocytes indicate expression of the GFP reporter and shGAPDH upstream. The lowest MOI (ratio of transducing lentiviral particles to cells) expressing adequate eGFP was 50 (Fig 6). This was used in all subsequent experiments. Determining the correct volume of lentiviral particles for effective transduction (stable adipocyte cell population with single shRNA integration) is essential given transduction efficiency varies widely depending on target cell type, duration of exposure to lentiviral particles and composition of transduction medium.

#### UCP1-shRNA expression in CL316, 243 treated cells

**Fig 7:**
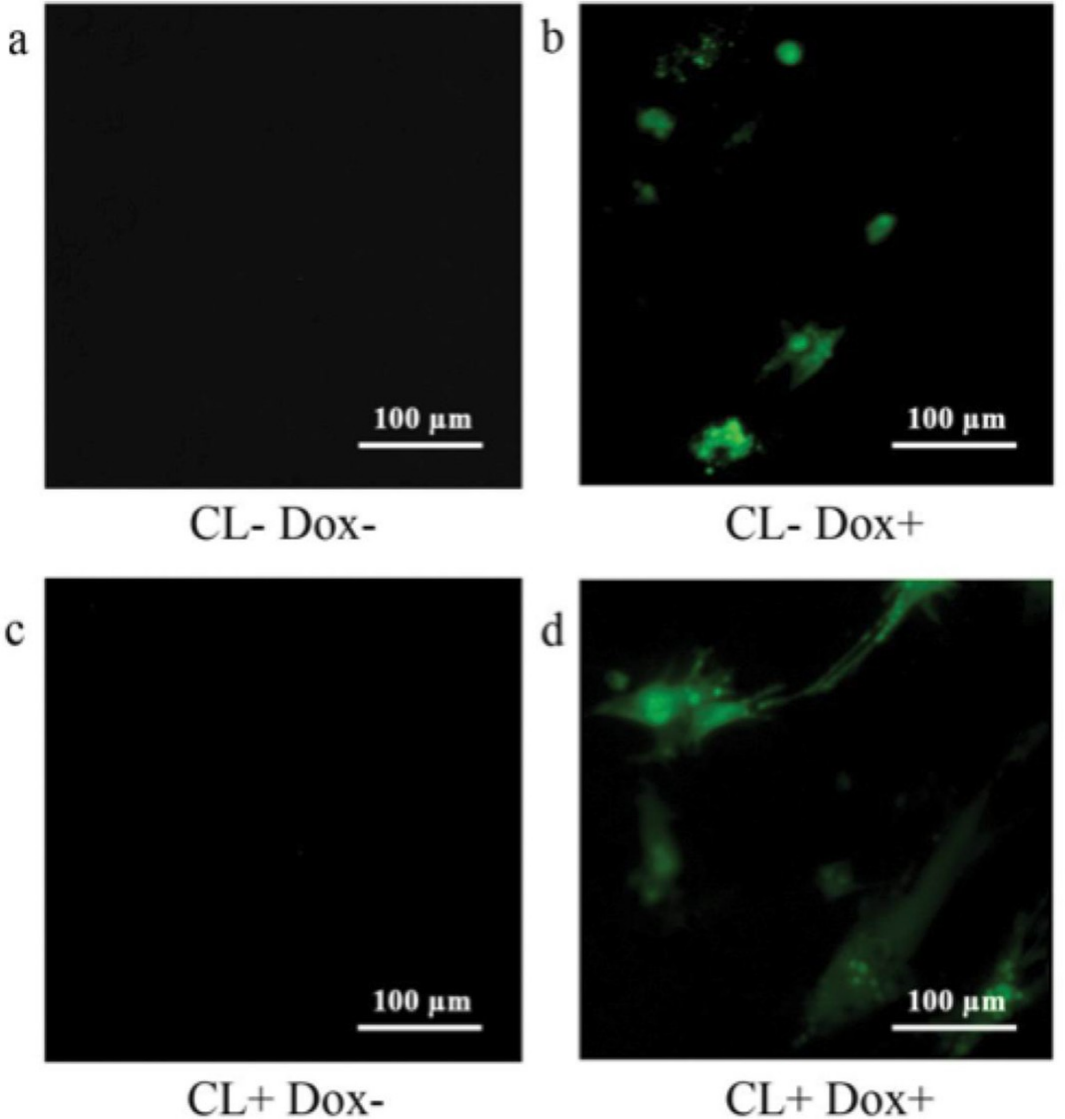
Cultured adipocytes transfected with lentivirus. (a) Control (CL-Dox-), (b) Control with lentivirus switched on (CL-Dox+), (c) CL treated (CL+ Dox-), (d) CL treated adipocytes with lentivirus switched on (CL+Dox+).

The next step was to determine the effects of UCP1-shRNA on UCP1 function in browned adipocytes from WAT. Cells were induced to differentiate to adipocytes and treated with CL316, 243 before transduction with the lentiviral vector. Cells were treated with doxycycline (700ng/ml) 24 post-transfection and then cultured for 24 hours before imaging. GFP was detected in wells treated with doxycycline indicating the lentivirus successfully expressed eGFP as a reporter gene and UCP1-shRNA gene downstream (Fig 7).

**Fig 8:**
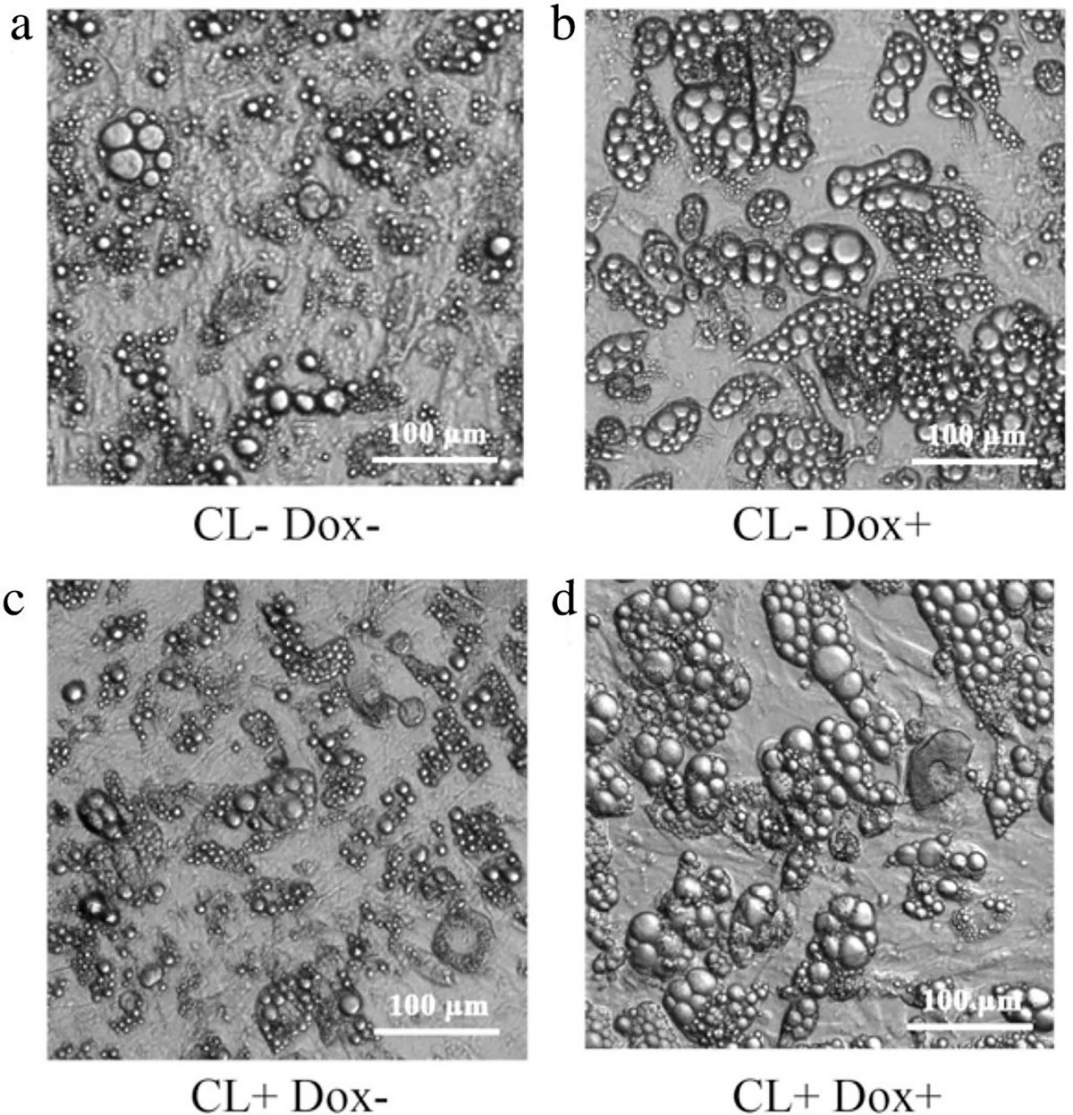
Cultured adipocytes transfected with lentivirus. Investigating effects of CL and UCP1-shRNA on primary beige adipocytes.

Next cell morphology was examined. Adipocytes treated with CL316, 243 contained smaller lipid droplets (a vs c, b vs d) suggesting unilocular to multilocular transformation during WAT browning. Compared to control (a), untreated adipocytes in the presence of doxycycline (b) showed larger lipid droplets, suggesting UCP1-shRNA inhibits background browning. In (d) the lentiviral UCP1-shRNA system is switched on in CL316, 243 treated cells. These cells contain larger lipid droplets heterogeneous in size, unlike the classic browning ‘multilocular’ appearance identified in (c) (Fig 8).

#### Effect of UCP1-shRNA on thermogenic gene expression

**Fig 9:**
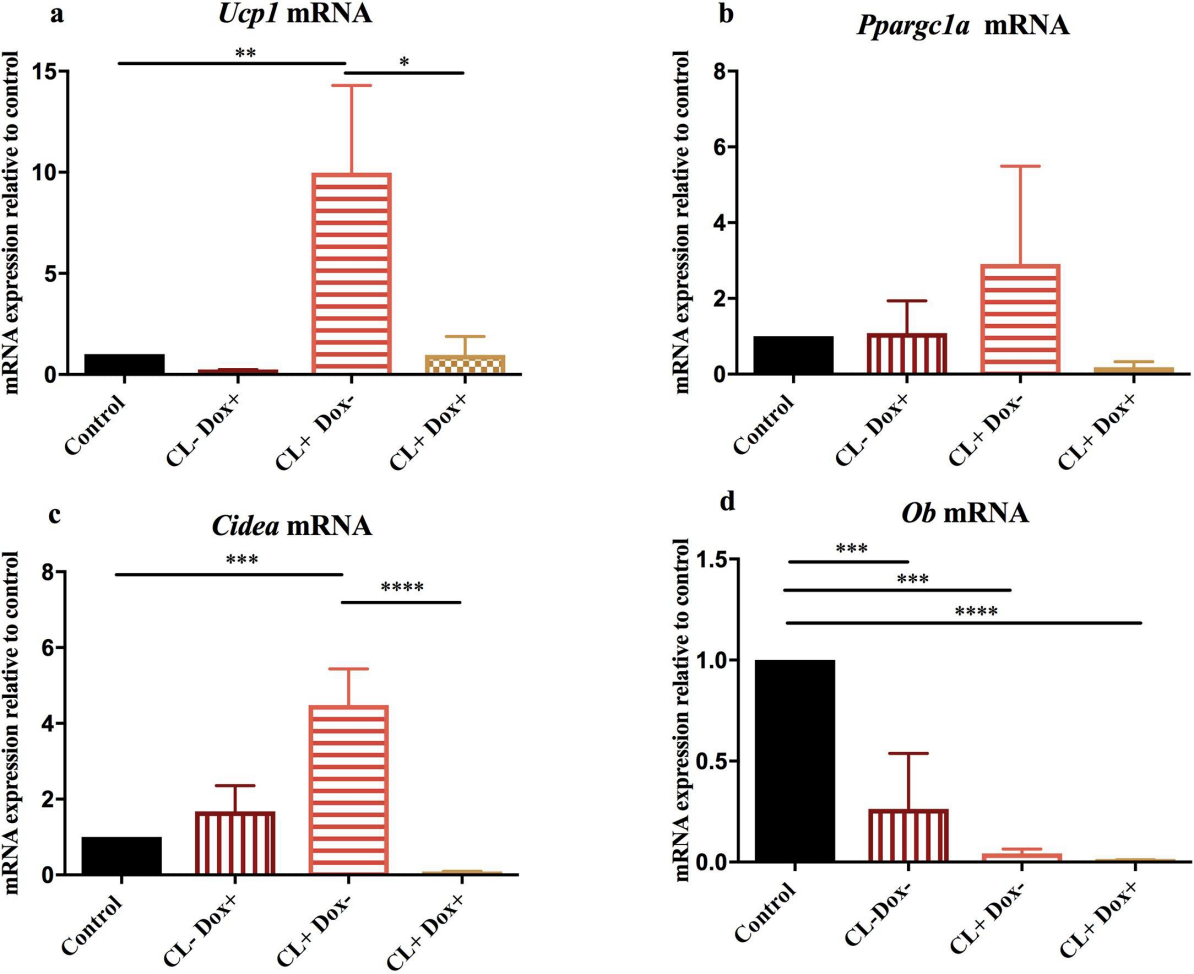
Relative amounts of *Ucp1*, *Ppargc1a*, *Cidea* and *Ob* mRNA in primary beige adipocytes. Adipocytes were transfected with lentivirus expressing shRNA-UCP1 and grown under control conditions (± Dox) or treated with CL316, 243 (± Dox). Data is normalized to corresponding 18S values. n = g12 *p<0.05, **p<0.01; ***p<0.001, ****p<0.0001, one-way ANOVA.

BAT thermogenesis is dependent on the induction of specific genes responsible for driving BAT development, transmission of sympathetic nervous signalling and mitochondrial uncoupling [18]. mRNA expression of *Ucp1*, the key hallmark of brown adipocytes, increased by 10-fold (p = 0.009) with the addition of CL316,243. Induction of UCP1-shRNA (CL+ Dox+) decreased *Ucp1* expression significantly such that it was at control levels. In untreated cells UCP1-shRNA reduced *Ucp1* expression to 24% of control levels suggesting silencing of background thermogenic activity (Fig 9A).

The master adipogenic factor PPARγ coactivator-1α (PGC-1α, encoded by *Ppargc1a*) controls expression of brown / beige fat genes, including *Ucp1* and is a dominant regulator of mitochondrial biogenesis, oxidative metabolism and thermogenesis [19]. *Cidea* is predominantly expressed in brown fat and is thought to regulate UCP1 activity [20]. Similar to the trajectory of *Ucp1* expression, *Ppargc1a*, and *Cidea* expression increases in CL316,243 treated cells and reduced below-control levels when UCP1-shRNA was induced. However unlike *Ucp1* expression, the addition of doxycycline in untreated control cells does not decrease expression below control levels (Fig 9B and 9C).

To gain further insight into the effects of UCP1-shRNA on adipocytes we measured the expression of *Ob* (Leptin), a hallmark of white adipocytes. CL316,243 treated cells (with UCP1-shRNA switched on and off), exhibit relatively low levels amounting to 0.04% and 0.01% of control levels respectively, possibly due to the effect of browning decreasing white adipocytes (Fig 9D).

Having shown the ability of the lentiviral shRNA system to induce morphological and genetic changes, the lentiviral UCP1-shRNA platform was tested on a functional level.

#### Effect of UCP1-shRNA on mitochondrial respiration of beige adipocytes

**Fig 10:**
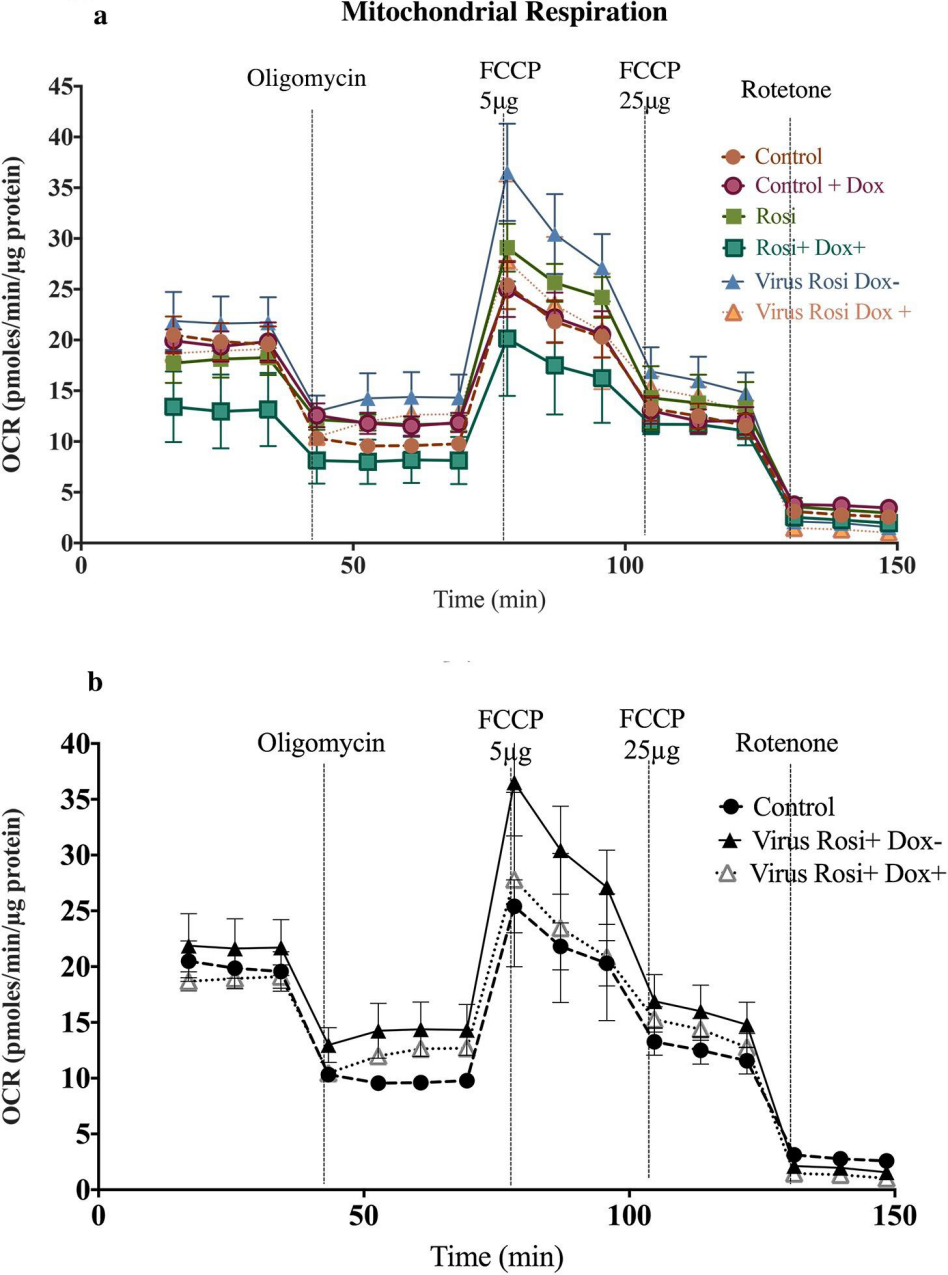
Mitochondrial stress test performed on UCP1-shRNA treated beige adipocytes. (a) Time course of oxygen consumption rate in the following groups: Control ± doxycycline, Rosiglitazone treated cells ± dox, Rosiglitazone viral-transfected cells ± dox. Data are represented as mean ± SEM; n = 20. Effect of doxycycline and lentivirus on respiratory measures in S3 Fig. (b) OCR in differentiated beige adipocytes in: control and Rosiglitazone viral-transfected cells ± dox. n = 9 *p<0.05, **p<0.01; ***p<0.001, ****p<0.0001, one-way ANOVA.

**Fig 11:**
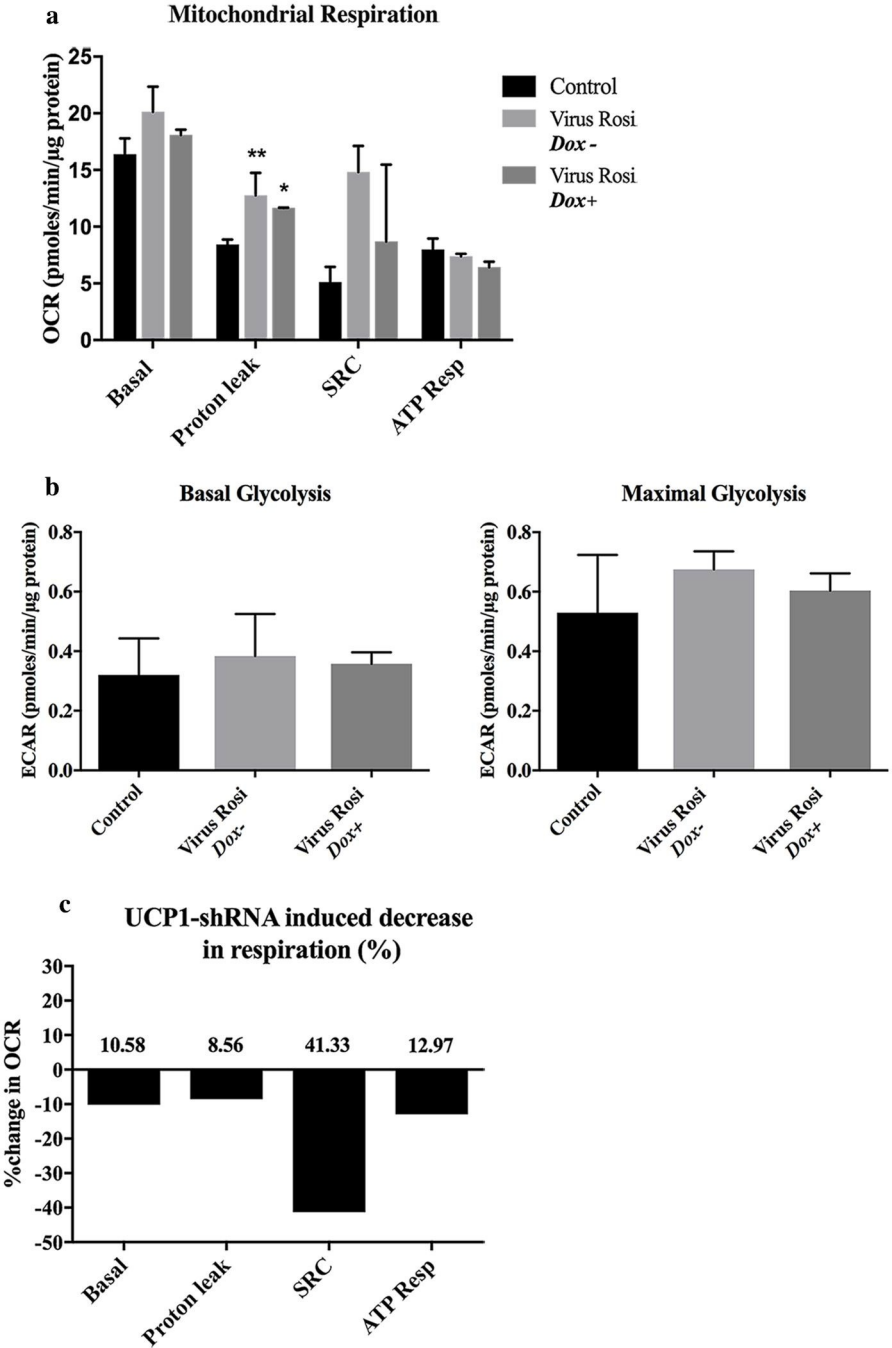
Parameters of mitochondrial respiration reduced on induction of UCP1-shRNA. (a) Derived parameters of mitochondrial respiration across control and Rosiglitazone viral-transfected cells ± dox. (b) Basal and maximal glycolysis. (c) Percentage decrease in respiration, induced with UCP1-shRNA.

With the addition of rosiglitazone basal respiration, spare respiratory capacity and proton leak increased compared to control, consistent with previous results. The only significant increase was proton leak (p = 0.006). Doxycycline was added to treated cells and mitochondrial stress test performed 48 hours later (Fig 10). Upon induction of UCP1-shRNA all four parameters of mitochondrial respiration decreased (Fig 11).

## Discussion

Few reports have quantitatively assessed the energy expenditure of browned white adipocytes. This study demonstrates UCP1 function can be manipulated by lentivirus in primary adipocytes. This provides a system for future studies exploring potential regulators of UCP1 activity and cellular metabolism. Our results show under maximal stimulation rosiglitazone and CL316, 243 can increases energy expenditure by 3–3.5-fold. CL316,243 significantly increased glycolytic rates as assessed by ECAR—approximately doubling both basal and maximal rates. Cypess and Weiner [21] recently demonstrated adrenergic signalling of BAT consumes fatty acids and glucose.

Limited trials of browning agents have been performed in humans or on human tissue, partly due to the paucity of non-invasive tools quantifying beige adipose tissue mass and activity [22]. The methods in the present study would be amenable to testing in human tissues. This methodology was recently illustrated by Bugge and Dib [16]. Alternatively adipocytes could be differentiated from preadipocytes isolated from human adipose tissue samples, so agents promoting browning could be evaluated in a cell culture system [23].

Our results demonstrate increased mitochondrial respiration and importantly a decrease of oxygen consumption rate across all indicators of respiration upon silencing of UCP1. Thermogenic recruitment is a concerted process involving an increase in functional mitochondrial UCP1 content, cell proliferation and mitochondrial biogenesis [24].

This present study quantifies the thermogenic contribution of CL316, 243, a specific β3-agonists. CL316,243 treatment resulted in an almost 10-fold increase in *Ucp1* mRNA, but only a 1.47 fold increase in UCP1 protein and a 1.65 fold elevation in proton-leak dependent respiration. Here we see an inconsistency between elevations in the various parameters of thermogenesis. Although *Ucp1* mRNA indicates tissue responsiveness to a stimulus, it may give inadequate conclusions for the metabolic significance of recruited adipose tissue. Indeed Nedergaard and Cannon [24] assert UCP1 protein is the actual heat-producing entity and offers a better indication of thermogenic capacity. We propose using the Seahorse XF Analyzer to measure the thermogenic capacity of CL316, 243 from a functional point of view. Indeed phase 2 clinical trials with β3-adrenergic agonists demonstrate improved glucose disposal, decreased plasma triglycerides and increased RMR [25–28].

Application of this tool to in vivo testing would help to address key questions in adipocyte biology. The virus could be administered via bilateral injection into fat depots and regulated by feeding mice doxycycline. The physiological relevance of beige fat has been questioned recently. Nedergaard and Cannon [24] report the relative contribution of beige adipose tissue to total thermogenic capacity is marginal compared to classical BAT; as expression of *Ucp1* mRNA and protein is at least an order of magnitude lower in beige cells. However obese humans have orders of magnitude more WAT than BAT. By silencing UCP1 in specific WAT depots, resulting changes in whole-body energy balance and glucose and lipid metabolism can provide a clearer picture of whole-body and depot specific effects on metabolism.

The inducible UCP1-knockdown technology provides a means to analyse the relative physiological importance of classical BAT verses beige, a difficult task as many agents affect both types of fat cells. Currently the CRE-LoxP recombination system achieves adipocyte specific targeting (Adpn-Cre, Fab4-Cre, Adpn-CreBAC), BAT selective targeting (Myf-cre, pax7-cre) and mature thermogenic adipocytes (UCP1-cre) [29–31]. Yet there is a lack of a robust model system to elucidate the individual contributions of the two distinct types of brown cells. With this technology, UCP1 can be silenced in adipose depots as determined by the researcher, an essential control as different depots possess varying capacity of browning with varying levels of inducible BAT [32]. With greater specificity, the contribution to energy metabolism of individual depots can be quantified and compared.

The inducible lentiviral shRNA method of genetic alteration overcomes limitations of the Cre-model surrounding expression at the correct level, time and place. Recombination can occur in 'non-fat tissue' complicating interpretation of adipose phenotype. The injectable shRNA enables study of expression and function of UCP1 solely in the adipose depot injected. Secondly the regulatory feature, controlled by the presence of the effector substance doxycycline, circumvents issues of recombination at embryogenesis affecting mice development and resulting adult phenotype.

Indeed a browning hierarchy exists where classical BAT is preferentially browned over WAT [33]. However, paucity of classical BAT may lead to increased sympathetic input to WAT, promoting formation of beige fat at a sufficient level to restore total thermogenesis [34, 35]. A mirror image to this hyperactive beige tissue, was seen in beige-knockout 'Adipo-PRDM16' mice which displayed a compensatory increase in BAT activity [10]. Applying the lentiviral UCP1-shRNA tool to brown and beige depots independently can help elucidate the complexity of systemic BAT-WAT crosstalk and compensatory interactions between depots.

Our study is limited by cell culture conditions rather than *in vivo* testing. We have chosen to study thermogenesis in culture as many of the functional differences between brown and white adipocytes are retained. Classical BAT show high baseline levels of *Ucp1*, *PGC-1a* and other factors of a thermogenic gene program compared to WAT, and stimulation exhibits a robust activation of uncoupled respiration [36].

We have shown the lentiviral UCP1-shRNA platform suppresses *Ucp1* expression and its thermogenic ‘browning’ effects. UCP1-shRNA lentivirus decreased *Ucp1* mRNA by 90% in CL316,243 treated adipocytes and 76% under physiological 'control' conditions. It is important to note experiments were conducted with the lentiviral tool switched on for 48 hours. It will be interesting to measure gene-silencing effects over time. Indeed a 90% decrease observed in *UCP1* mRNA may take time to manifest functionally, considering the half-life of UCP1 is widely variable between 30–72 hours [37].

## Conclusion

As expected, under stimulation of CL316, 243 and rosiglitazone, white adipocytes from the inguinal depot efficiently converted into thermogenic beige adipocytes. There was unilocular-to-multilocular transformation, and increased UCP1 protein. This was accompanied by physiologically relevant increases in mitochondrial respiration. Importantly these parameters decreased when UCP1 shRNA was delivered and then induced with doxycycline. This has two important ramifications—firstly UCP1 was responsible for the observed increases in thermogenic activity and mitochondrial respiration. Secondly the lentiviral UCP1 shRNA platform effectively suppresses *Ucp1* expression and thermogenic effects in adipocytes, stimulated with browning agents. Inducible UCP1-knockdown holds potential to interrogate the physiological relevance of recruitable 'beige' depots. This tool can shed light in the complex field of adipose biology—determine the proportional contribution of the two distinct brown adipocyte lineages to thermogenesis as well as provide insight into systemic BAT-WAT interactions.

## Supporting information

S1 Fig Inducible lentiviral shRNA vector encoding UCP-1. In the presence of doxycycline the constitutively active **Tet-On 3G** undergoes a conformational change, allowing it to bind to the **TRE3G** promoter. TRE3G is activated and expresses the gene of interest (shRNA). **eGFP—**enhanced green fluorescent protein; permits infected cells to be identified by fluorescence.
(TIF)

S2 Fig Adipocyte differentiation and treatment with rosiglitazone and CL 316,243 on day 1, 4, 7 post treatment. (TIF)

S3 Fig Effect of doxycycline and lentiviral vector on respiration. (a) Control ± doxycycline, (b) Rosiglitazone treated cells ± doxycycline (c) Lentiviral transfected cells in rosiglitazone treated cells ± doxycycline (d) Rosiglitazone treated cells ± lentivirus (e) Rosiglitazone treated cells plus doxycycline ± lentivirus. Data are represented as mean ± SEM; n = 20 *p<0.05, **p<0.01; ***p<0.001, ****p<0.0001, analysed by one-way ANOVA.
(TIF)

## Abbreviations

BAT: brown adipocytes
BMR: basal metabolic rate
CIDEA: cell death-inducing DFFA-like effector A
Dox: doxycycline
EE: energy expenditure
eGFP: enhanced green fluorescent protein
FCCP: carbonilcyanide *p*-triflouromethoxyphenylhydrazone
MOI: multiplicity of infection
Mouse EF-1α: mouse elongation factor-1 alpha
mRNA: micro RNA
Ob: obese gene (leptin)
PCG-1α: PPARγ coactivator—1α
PET 18F FDG: positron emission tomography 18F fluorodeoxyglucose
PPARγ: peroxisome proliferator-activated receptor gamma
PPDRM16: PR domain containing 16
Rosi: Rosiglitazone
shRNA: short hairpin RNA
TBP: TATA-Box Binding Protein
TBX1: T-box transcription factor 1
TRE3G: inducible promoter with tetracycline response elements
UCP1: uncoupling protein 1
WAT: white adipocytes
