# Supplementary figures and images for: Inducible UCP1 silencing: A lentiviral RNA-interference approach to quantify the contribution of beige fat to energy homeostasis
Source: PLoS One. 2019 Nov 21;14(11):e0223987. doi: 10.1371/journal.pone.0223987 (PMC6872148; doi:10.1371/journal.pone.0223987)

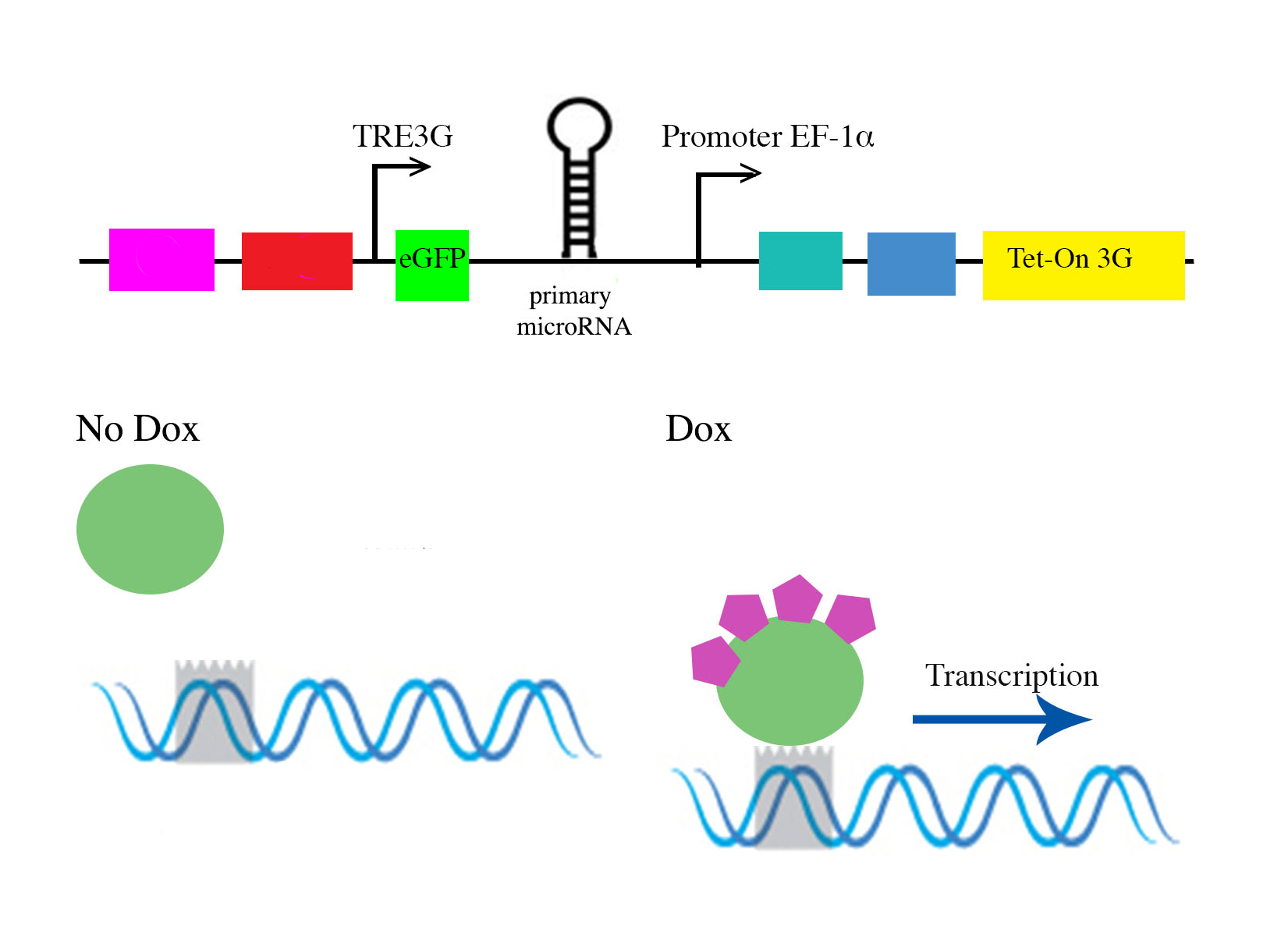

Supplement: S1 Fig — In the presence of doxycycline the constitutively active Tet-On 3G undergoes a conformational change, allowing it to bind to the TRE3G promoter. TRE3G is activated and expresses the gene of interest (shRNA). eGFP—enhanced green fluorescent protein; permits infected cells to be identified by fluorescence. (TIF) [file pone.0223987.s001.tif]

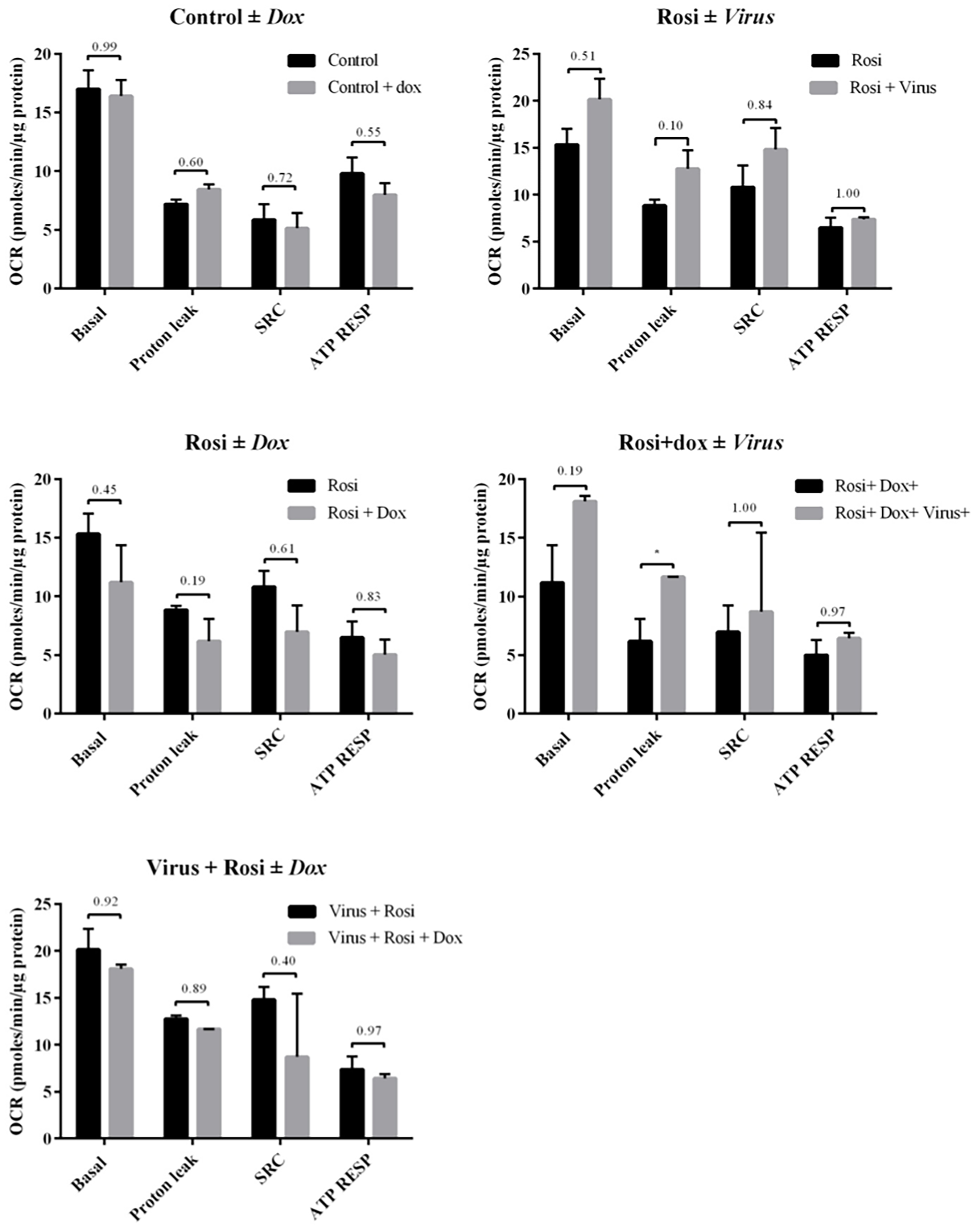

Supplement: S3 Fig — (a) Control ± doxycycline, (b) Rosiglitazone treated cells ± doxycycline (c) Lentiviral transfected cells in rosiglitazone treated cells ± doxycycline (d) Rosiglitazone treated cells ± lentivirus (e) Rosiglitazone treated cells plus doxycycline ± lentivirus. Data are represented as mean ± SEM; n = 20 *p<0.05, **p<0.01; ***p<0.001, ****p<0.0001, analysed by one-way ANOVA. (TIF) [file pone.0223987.s003.tif]
